# Post-thaw quality assessment of testicular fragments as a source of spermatogonial cells for surrogate production in the flatfish *Solea senegalensis*

**DOI:** 10.1007/s10695-023-01232-2

**Published:** 2023-08-29

**Authors:** Elsa Cabrita, Tiziana Pacchiarini, Elvira Fatsini, Carmen Sarasquete, María Paz Herráez

**Affiliations:** 1 Centre of Marine Sciences-CCMAR, University of Algarve, Campus Gambelas, 8005-139 Faro, Portugal; 2 Sea4tech, Incubadora de Alta Tecnología INCUBAZUL, Edificio Europa, Zona Franca de Cádiz, Cádiz, Spain; 3 Institute of Marine Science of Andalusia- ICMAN.CSIC, Av Republica Saharaui 2, 11510 Puerto Real, Cádiz, Spain; 4 Dept. Biologia Molecular, Facultad de Biologia, Universidad de León, 24071 León, Spain

**Keywords:** Cryopreservation, Spermatogonia, Testicular germ cells, Cell viability, DNA fragmentation

## Abstract

Cryopreservation of germ cells would facilitate the availability of cells at any time allowing the selection of donors and maintaining quality control for further applications such as transplantation and germline recovery. In the present study, we analyzed the efficiency of four cryopreservation protocols applied either to isolated cell suspensions or to testes fragments from Senegalese sole. In testes fragments, the quality of cryopreserved germ cells was analyzed in vitro in terms of cell recovery, integrity and viability, DNA integrity (fragmentation and apoptosis), and lipid peroxidation (malondialdehyde levels). Transplantation of cryopreserved germ cells was performed to check the capacity of cells to in vivo incorporate into the gonadal *primordium* of Senegalese sole early larval stages (6 days after hatching (dah), pelagic live), during metamorphosis (10 dah) and at post-metamorphic stages (16 dah and 20 dah, benthonic life). Protocols incorporating dimethyl sulfoxide (DMSO) as a cryoprotectant showed higher number of recovered spermatogonia, especially in samples cryopreserved with L-15 + DMSO (0.39 ± 0.18 × 10^6^ cells). Lipid peroxidation and DNA fragmentation were also significantly lower in this treatment compared with other treatments. An important increase in oxidation (MDA levels) was detected in samples containing glycerol as a cryoprotectant, reflected also in terms of DNA damage. Transplantation of L-15 + DMSO cryopreserved germ cells into larvae during early metamorphosis (10 dah, 5.2 mm) showed higher incorporation of cells (27.30 ± 5.27%) than other larval stages (lower than 11%). Cryopreservation of germ cells using testes fragments frozen with L-15 + DMSO was demonstrated to be a useful technique to store Senegalese sole germline.

**Supplementary Information:**

The online version contains supplementary material available at 10.1007/s10695-023-01232-2.

## Introduction

Germ cell transplantation has proved to be a valuable technique for studying processes related to spermatogenesis (cell proliferation, cell-to-cell interactions), restoring fertility in infertile individuals, or producing transgenic animals from genetically modified germ cells. It is considered a powerful tool in assisted reproductive strategies (Schlatt 2002) and its potential applications in fish reproductive biotechnology opened new research lines in cultured species and in the conservation of genetic resources of endangered species (Hagedorn et al. 2018). Transplantation models are also needed for studies on stem cell biology in marine fish, providing more in-depth knowledge of spermatological processes (Shulz et al. 2010), especially in species with reproductive dysfunctions. They are also important as in vivo methods for evaluating the functionality of cryopreserved spermatogonia.

Germ cell transplantation has been addressed in several fish including model species such as zebrafish (*Danio rerio*), loach (*Misgurnus anguillicaudatus*), and goldfish (*Carassius auratus*) (Marinović et al. 2019; Nóbrega et al. 2010); commercial species such as salmonids, Nile tilapia (*Oreochromis niloticus*), or common carp (*Cyprinus carpio*) (Franek et al. 2019; Lacerda et al. 2010; Takeuchi et al. 2004); and species for conservation purpose such as the Murray river rainbowfish (*Melanotaenia fluviatilis*), the brown trout (*Salmo trutta*), the Chinese sturgeon (*Acipenser sinensis*), or piracanjuba (*Brycon orbignyanus*) (de Siqueira-Silva et al. 2021; Fernández-Díez et al. 2012; Ye et al. 2017; Rivers et al. 2020a, 2020b). This technique can be also applied in species suffering reproductive dysfunctions in captivity or in those difficult to maintain due to their large size at maturation, such as the yellowtail (*Seriola quinqueradiata*) and bluefin tuna (*Thunnus orientalis*) (Morita et al. 2012; Yazawa et al. 2021). It has been also applied in some donor sturgeon species with late maturation where faster-maturing hosts can be used to produce donor progeny (Psenicka et al. 2015; Franek et al. 2022).

Most of the research developed used fresh isolated spermatogonia obtained after testes dissociation. Fewer studies used spermatogonia obtained from in vitro culture or cryopreserved, although these management techniques can be a useful tool and source for germ cell banks for conservation or production management. Regarding the use of cryopreserved cells, there are some concerns that need to be addressed related to in vitro and in vivo post-thaw quality that have been most of the time neglected.

In the present study, we developed and assessed a cryopreservation methodology for the cryostorage of testicular fragments containing Senegalese sole germ cells and we analyzed their in vitro post-thaw quality, as well as their ability to colonize host gonads after larval intraspecific transplantation. Senegalese sole undergoes a complex metamorphic process during larval development, involving the final stages of organogenesis, including genital ridge germ cell colonization, formation of the undifferentiated gonads, and several morphological and physiological changes (Pacchiarini et al. 2013; Sarasquete et al. 2019). Its high commercial value represents a good candidate for the application of this technology since some reproductive constraints in captivity have been identified in F1 breeders (Morais et al. 2016). The availability of cells from the germline and the application of reproductive strategies based on germ cell transplantation could counteract some of these reproductive problems from a biological and productive point of view, especially if cryopreserved germ cells could be used in transplantation studies and if a host candidate is available (Pacchiarini et al. 2014; Zhou et al. 2021).

Several procedures need to be attuned to establish a successful donor germ lineage in the host gonad after transplantation. Some of them, such as cell isolation, cell labeling, choice of the proper host, or preparation of the recipient gonad, have been addressed in several studies, as reviewed by Lacerda et al. (2013). As mentioned before, cryopreservation protocols for spermatogonia of some species have been attempted. Nevertheless, not much attention has been given to the evaluation of the quality of the transplanted germ cells, obtained either from fresh or cryopreserved tissues. This represents a crucial factor since it has been demonstrated that the use of cryopreservation can produce cell damage affecting recovery and cell incorporation (Marinović et al. 2019; Zupa et al. 2020), or even some defects in gametogenesis or cell proliferation (Whelan et al. 2022; Xie et al. 2020).

Cryopreservation of germ cells would facilitate the availability of cells at any time, allowing transplantation when the recipient species is not synchronized with the donor. It would also be of great use in endangered species or specimens captured overseas. Transplantation procedures would be simplified since donor species can be previously selected and the germ cells stored, maintaining control of sample quality. A cryopreservation procedure for isolated primordial germ cells (PGCs) was applied in rainbow trout (Kobayashi et al. 2007), though the characterization of these cells was neglected. Cryopreserved germ cells were also used in Nile tilapia by Lacerda et al. (2010), demonstrating promising results of cell functionality, but a certain delay in testes colonization in comparison with fresh cells. These data are encouraging, but the establishment of post-thaw quality analysis in cryopreservation protocols seems to be crucial for studies in germ cell transplantation.

## Material and methods

All chemicals, unless otherwise stated, were purchased from Sigma-Aldrich and were reagent grade or higher.

### Testes and isolated cells

Twelve-month-old immature fish (length: 12.54 ± 1.3 cm; weight: 27.63 ± 2.8 g) were acquired from a fish farm and euthanized with a lethal dose of phenoxyethanol (2000 ppm). Testes were removed by surgical incision and maintained in PBS or L-15 media (Leibovitz) until processed (no longer than 15 min). To obtain isolated germ cells, testes were sliced with surgical micro scissors (Vannas, Quirumed) and the small fragments were incubated in 1500 μL of 0.25% trypsin in PBS + 0.5% FBS + 200 units DNase-I for 2 h at 22 °C with gentle shaking (350 rpm). Cells were pipetted to separate clumps every half hour. Trypsin treatment was stopped by adding 500 μL of PBS or L-15 plus 0.5% FBS to the cell suspension followed by filtration with a 80-μm mesh and washed twice by centrifugation (400 *g*× 10 min 15 °C) to eliminate the trypsin. The cells were maintained at 4 °C in 300 μL PBS or L-15 depending on the cryopreservation protocol followed. To obtain fragments, each testis was cut into 3 or 4 small pieces of approximately 1 mm^3^ and kept in PBS or L-15 at 4 °C.

### Cryopreservation of cell suspensions vs testes fragments

**Fig. 1:**
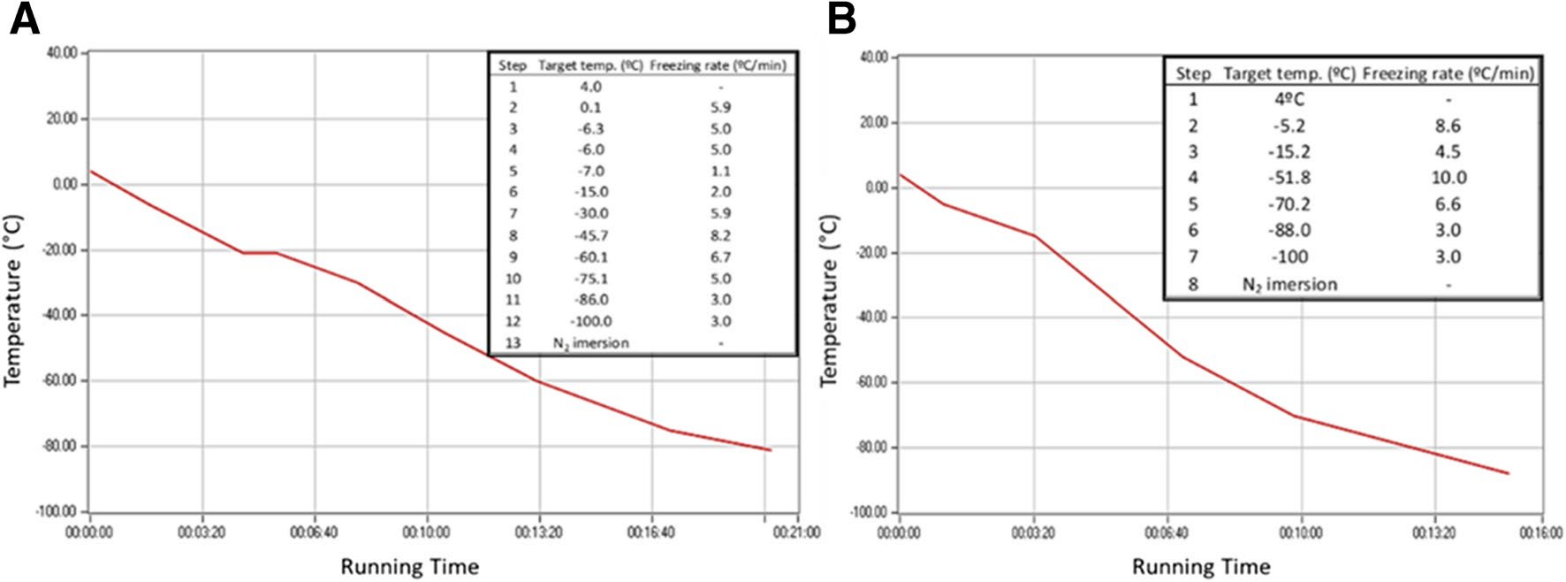
Freezing conditions used to cryopreserve testicular germ cells both in cryovials (**A**) and 0.5 mL French straws (**B**). Cryovials were used to freeze testes fragments containing spermatogonia and straws were used to freeze cell suspension also containing these cells

For this experiment, 35 fish were used, collecting one testis from each male for cell isolation and the other one for gonadal tissue fragments. Two cryopreservation procedures were tested using cell suspensions obtained after testes trypsinization (*n*=35), as described above, or small testes fragments (*n*=35). Cellular suspensions and testes fragments were cryopreserved in PBS or L-15 based media supplemented with 0.5% BSA and 5.5 mM glucose with 1.5 M dimethyl sulfoxide (DMSO) or 1.5 M glycerol (modified from Kobayashi et al. 2007). For all treatments, isolated cells were loaded in 0.5 mL French straws (*n*=10) (IMV, France) and testes fragments were stored in cryovials containing 500 μL of freezing media (10–12 fragments per cryovial) (*n*=8) and left to equilibrate for 15 min at 4 °C. Freezing was performed using a portable programmed biofreezer (Asymptote EF600, Grant Instruments, Cambridge, UK) following the steps described in Fig. 1. The freezing rates were settled in the biofreezer according to a previous protocol (Cabrita et al. 2010), where samples were frozen 2 cm above liquid nitrogen and the temperature was registered using a thermocouple. Freezing rates are expressed in Fig. 1A for cryovials and Fig. 1B for straws. Thawing was carried out in a water bath at 25 °C for 30 s for the straws and 40 °C for 140 s for the cryovials. Thawed testes fragments were dissociated as previously described to obtain cell suspensions. Plasma membrane integrity was analyzed in both cryopreservation treatments (isolated cells *vs* testes fragments) after thawing using PI/SYBR-14 double staining (Invitrogen, Madrid, Spain).

### In vitro cell analysis of post-thaw testes fragments

To perform a more exhaustive analysis of cell quality after thawing, cell recovery, plasma membrane integrity and viability, oxidative stress, DNA integrity, and cell apoptosis were performed in cells obtained from post-thaw fragments, following the protocols previously described. In this experiment, testes from 40 male juveniles (80 testes) were cut into pieces as previously described, loaded in 40 cryovials, and frozen with the above four solutions (10 vials per solution), following the protocol for freezing-thawing previously described.

To perform the evaluation of the cryopreservation protocols, frozen/thawed testes fragments were dissociated according to the protocol described, and cells were resuspended in PBS or L-15 media.

### Germ cell recovery

Spermatogonia differential cell counting (SPG) in the cell suspension was performed with a Neubauer chamber in a fluorescence microscope (Nikon Eclipse E400; Nikon, Japan) with a 40× objective using DAPI stain. SPGs were identified by their specific morphology (large and granular nucleus) according to Takeuchi et al. (2009)**.** The number of recovered spermatogonia after dissociation of frozen/thawed testes was registered in 8 samples per treatment.

### Plasma membrane integrity and cell viability

Plasma membrane integrity was analyzed with the live/dead sperm viability kit (Invitrogen, Spain). Cells were incubated in the dark for 5 min with 0.25 μM SYBR-14 plus 5 min with 24 μM propidium iodide (PI) at 4 °C. At least 100 cells per sample were observed (40×) with a fluorescence microscope (Nikon Eclipse E400; Nikon, Japan) with a blue excitation filter (450–480 nm). Cells staining red were classified as membrane permeable to PI whereas green cells were classified as non-permeable to PI. Plasma membrane integrity was calculated from the number of cells counted as non-permeable to PI with respect to the total number of cells. At least 100 cells were counted in triplicate.

Cell viability (metabolic active cells) was determined using calcein AM (Invitrogen, Spain). Briefly, 50 μL of cell suspension (5 × 10^4^ cells) from each treatment (PBS + glycerol; PBS + DMSO; L-15 + glycerol and L-15 + DMSO) was incubated with 50 μL of 2 μM calcein AM (final concentration 1 μM) for 30 min and fluorescence emission was determined by fluorometry (Infinite 200 PRO, TECAN, Spain) after excitation at 515 nm. In viable cells, the non-fluorescent calcein was converted to green-fluorescent calcein and retained in intact cells, after acetoxymethyl ester hydrolysis by intracellular esterase. Higher absorbance levels indicated a higher number of cells with the intact plasma membrane and esterase activity (Kaya et al. 2003).

A total of 10 samples per treatment were analyzed.

### Oxidative stress

Oxidative stress was determined by lipid peroxidation through the determination of malondialdehyde (MDA) concentration, a subproduct of lipid peroxidation, using a colorimetric assay (Bioxytech LPO-586™ kit, OxisResearch™, USA) and following the protocol described by Martínez-Páramo et al. (2012), adapted for germ cells. Briefly, 100 μL of cell suspension was sonicated for 6 s and incubated in a 200 μM sodium ascorbate solution containing 40 μM FeSO_4_ for 30 min at 37 °C in the dark to induce MDA release. After that, reagents provided by the kit were added to 100 μL of the cell suspension, following the manufacturer’s instructions, and the samples were incubated for 1 h at 45 °C in the dark. Samples were then centrifuged at 10,000 *g* for 10 min at 4 °C, and 200 μL of supernatants was transferred to a 96-well flat-bottom transparent plate (Nunc, Denmark). Absorbance was read using a microplate reader at 586 nm (Infinite 200 PRO, TECAN, Spain) and MDA concentrations were calculated from a standard curve (0 to 30 μM MDA) and presented as μM of MDA × 10^4^ germ cells. Each sample was processed in triplicate. A total of 10 samples were analyzed.

### DNA integrity

DNA fragmentation was determined using the comet assay method (single-cell gel electrophoresis). Cells from each treatment (5 × 10^4^ cells) were embedded in agarose slides according to Cabrita et al. (2005), with some modifications. For cell lysis, DNA denaturation, and electrophoresis, buffers and solutions were freshly prepared with milli-Q water. The slides were placed in a Coplin jar containing lysis solution (2.5 M NaCl, 100 mM Na_2_-EDTA, 10 mM Tris, 1% Triton X-100, 1% lauryl sarcosine) for 1 h at 4 °C. To decondense the DNA, dithiothreitol was added to the lysis buffer to a final concentration of 10 mM and the slides were immersed for 30 min at 4 °C. After lysis, the slides were placed horizontally in an electrophoresis cube (BioRad, Spain) filled with electrophoresis solution (0.3 M NaOH, 1 mM Na_2_-EDTA, pH 12) for 30 min at 4 °C to allow the DNA to unwind. Electrophoresis was then conducted for 10 min at 25 V and 300 mA at 4 °C. Amperage was controlled by adjusting the volume of the electrophoresis buffer before the onset of the procedure. After electrophoresis, the slides were drained and placed in a Coplin jar with neutralizing solution (0.4 M Tris, pH 7.5) for 5 min at 4 °C. This operation was carried out twice to ensure the elimination of all alkalis and detergents. The slides were left to drain and sample fixation was performed by immersing the slides in a pure ethanol solution for 3 min. The slides were then left to drain in the air and stored protected from light and dust.

For comet visualization, 25 μL of propidium iodide (0.1 mM) was pipetted into the sample and covered with a coverslip. The samples were observed in an epifluorescence microscope (Leica DM 2000, Germany) with an excitation filter of 510–560 nm and a barrier filter of 590 nm. Each slide was analyzed randomly selecting several fields for image recording. Approximately 80 spermatogonia from each slide were recorded with a digital camera (Leica DFC420C) and Leica Application Suite software. Comet analysis was performed with the imaging system Komet software v6.0 (Andor Technology, Belfast, Ireland). For each cell analyzed, the percentage of tail DNA (% DNA_t_) was used to characterize fresh and cryopreserved spermatogonia. This parameter has been described elsewhere (Cabrita et al. 2005). A total of 10 cryovials per treatment were processed.

### Cell apoptosis

Apoptotic cells were identified in 5-μm slide sections obtained from post-thaw fragments and from fresh fragments using the in situ cell death detection kit (Roche, Barcelona, Spain), which identifies DNA damage at 3′-OH endings. The slides were treated with proteinase K (10 μg/mL in 10 mM Tris/HCl, pH 7.4) for 30 min at 37 °C to permeabilize cells, washed with PBS, and dried. Each slide was incubated with 5 μL of enzymatic solution and 45 μL of labeling solution (Br-dUTP) for 1 h at 37 °C in the dark. After rising the slides twice with rising buffer, the slides were incubated in the dark with anti-BrDU FITC for 30 min at RT according to the manufacturer’s instructions. Two controls were used: a positive control consisting of slides treated with DNase I (3 U/mL in 50 mM Tris/HCl, pH 7.5) for 10 min prior to labeling and a negative control consisting of slides incubated without the enzymatic solution. All the slides were mounted with Vectashield (Vector Laboratories, USA) and observed in a fluorescent microscope (LEICA DM 2000, Germany) with 450–490-nm excitation and 515-nm emission. Images were recorded and analyzed with a digital camera (LEICA DFC 420C, Germany) and the software LEICA Application Suite.

### In vivo cell functionality—intraspecific transplantation

For the rearing of host larvae, four *Solea senegalensis* spawns were incubated at 22 ± 1 °C in 4 circular tanks (400 L) with an aeration supply. Larvae hatched 2 days later and feeding started 2 days after hatching. Enriched rotifers (Red Pepper, Bernaqua, Belgium) were added twice a day to the tanks and from day 8 until the end of the experiment (day 42) the larvae were fed ad libitum with enriched *Artemia* nauplii (Red Pepper). *Nanocloropsis oculata* and *Isochrysis galbana* were supplied to all tanks (green water method) 2 days after hatching until the end of the experiment.

For the transplantation of post-thaw isolated germ cells, testes from 32 males were frozen in fragments as described above with L-15 + DMSO. Two cryovials were thawed (water bath, 40 °C, 140 s) and fragments dissociated on each day of microinjection. For cell visualization, the suspension was stained with a PKH26 kit, according to the manufacturer’s protocol (Merck, Portugal). The dye allowed the visualization of the cells for long periods after microinjection. The cells were maintained at 4 °C in L-15 at the desired concentration for 1 h before microinjection.

Transplantation experiments were performed on larvae at 6, 10, 16, and 20 days after hatching (dah) (length size: 4.0 ± 0.21, 5.2 ± 0.17, 7.3 ± 0.84, and 8.6 ± 0.20 mm)), which corresponded at the rearing temperature (22 ± 1 °C) to pelagic life, the beginning of metamorphosis, end of metamorphosis, and post-metamorphic life, respectively (Pacchiarini et al. 2013). Labeled cells (13.4 nL) were microinjected into the peritoneal cavity of 120 larvae (30 larvae per spawn, four spawns) at each stage of development. Prior to microinjection, transplantation needles were prepared by pulling glass capillaries (GD-1) (Narishige, Japan) using a magnetic puller (PN-31, Narishige, Japan). The needle tips were sharpened with a grinder (EG-400, Narishige, Japan) adapting the needle opening diameter (from 40 to 60 μm) and angle to the larvae skin resistance. The larvae were anesthetized with 0.0075% MS222 (tricaine) in seawater for 1 min. After microinjection and anesthesia recovery, they were separated into four batches and incubated in small incubation units (1 L) at 20 ± 1 °C for 3 weeks. A control group (no microinjection) from each spawn was incubated under the same conditions. Each day the larvae were counted, and dead individuals were recorded and removed from the incubation units. In order to determine Senegalese sole sensitivity to the microinjection procedure, survival rates were calculated for 1, 7, 14, and 21 days after microinjection. The larvae were fed according to the previously described procedure. At the end of the experiment (3 weeks after microinjection), larvae (control and microinjected) were fixed in Carnoy (60% methanol, 30% chloroform, 10% glacial acetic acid), to avoid fixative autofluorescence, and observed in a fluorescence microscope excited with a 450–480-nm filter (LEICA, Germany). In parallel, each larva was also observed with brightfield. Images were recorded with a digital camera and the Application Suite software (LEICA, Germany). The percentage of larvae incorporating fluorescent spermatogonia was assessed by counting larvae with stained germ cells in comparison with the total number of larvae microinjected in each batch. No red fluorescence was observed in the control groups.

### Statistical analysis

The statistical analysis was performed with SPSS Statistics 25.0 software for windows. All results are presented as mean values plus standard deviation. The Shapiro–Wilk test was used to analyze data normality and logarithmic transformation was performed when data were not normally distributed. For all cases, the level of significance was considered with a *p*-value < 0.05. A one-way ANOVA was applied followed by a Student-Newman-Keuls (SNK) test (*p* < 0.05) to identify significant differences between treatments for each descriptor.

## Results

### Cryopreservation of cell suspensions vs testes fragments

**Fig. 2:**
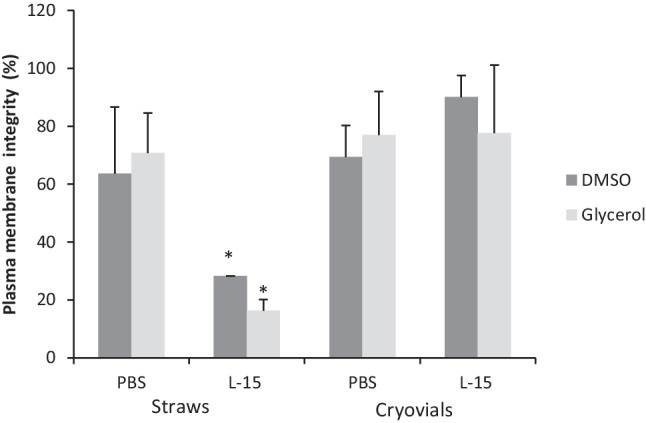
Plasma membrane integrity obtained for germ cell suspensions frozen in 0.5-mL straws and testes fragments frozen in cryovials both in phosphate buffer (PBS) or Leibovitz media (L-15) containing dimethyl sulfoxide (DMSO) or glycerol. Significant differences were signed with asterisks (*) (mean values ± SD, *n*=8, *p* ≤ 0.05)

The cryopreservation of germ cell suspensions and testes fragments provided rates of cell integrity ranging between 90.05 ± 7.5% (testes fragments frozen with L-15 + DMSO) and 16.3 ± 3.8% (isolated cells frozen with L-15 + glycerol). No differences were observed according to the used cryoprotectants, with the exception of cells frozen in straws with DMSO or glycerol, showing glycerol worst results. More than 70% of cells dissociated from post/thaw testes fragments presented plasma membrane integrity with any of the tested solutions. Germ cell suspensions frozen in L-15 extender, with both DMSO and glycerol, reached significantly lower cell integrity rates than the other treatments (Fig. 2).

### Cryopreservation of testes fragments: evaluation of thawed cells

**Fig. 3:**
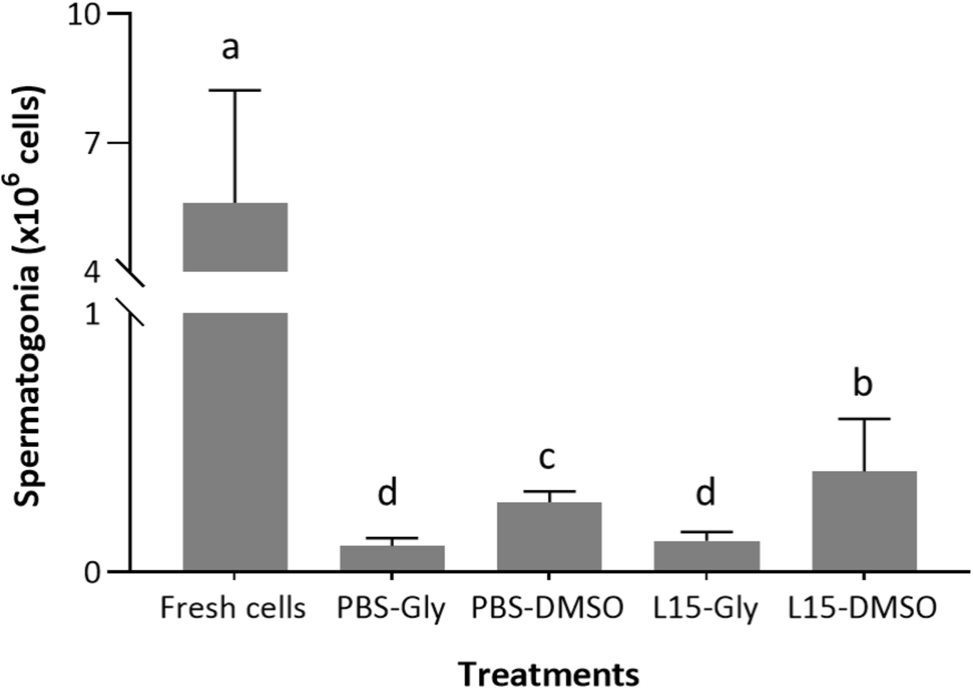
Recovered spermatogonia after cryopreservation of testes fragments in PBS or L-15 media incorporating DMSO or glycerol. Isolated cells were obtained after testes dissociation. Significant differences between treatments are signed by different letters (mean values ± SD, *n*=10, *p* ≤ 0.05)

The use of DMSO allowed the recovery of a significantly higher number of spermatogonia per cryovial (between 0.39 ± 0.18 × 10^6^ cells in L-15 + DMSO to 0.27 ± 0.04 × 10^6^ cells in PBS + DMSO) than glycerol treatments (less than 0.12 × 10^6^ cells) (Fig. 3).

**Fig. 4:**
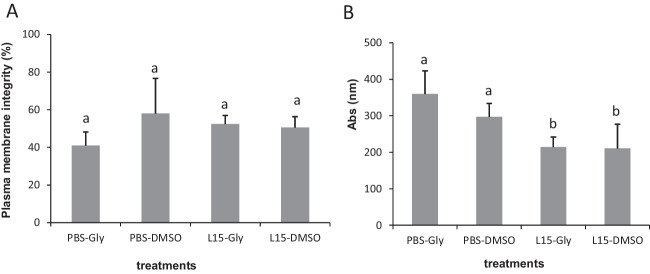
Plasma membrane integrity determined using the dual stain PI/SYBR-14 (A) and viable cells (B) determined by calcein AM. Higher absorbance corresponds to an increase in the esterase activity of intact cells. Cells were obtained after the dissociation of testes fragments cryopreserved in PBS or L-15 media incorporating DMSO or glycerol. Significant differences between treatments are signed by different letters (mean values ± SD, *n*=10, *p* ≤ 0.05)

Cells obtained by dissociation of thawed testes fragments using different extenders and cryoprotectants showed 57.96 ± 26.31% of plasma membrane integrity for fragments frozen with PBS + DMSO and 40.95 ± 10.22% for those frozen with PBS+glycerol, although there were no significant differences between treatments (Fig. 4A). The use of calcein to detect viable cells revealed a significant effect of the extender, a higher rate of metabolic active cells being recorded in samples frozen with PBS than with L-15 (Fig. 4B).

**Fig. 5:**
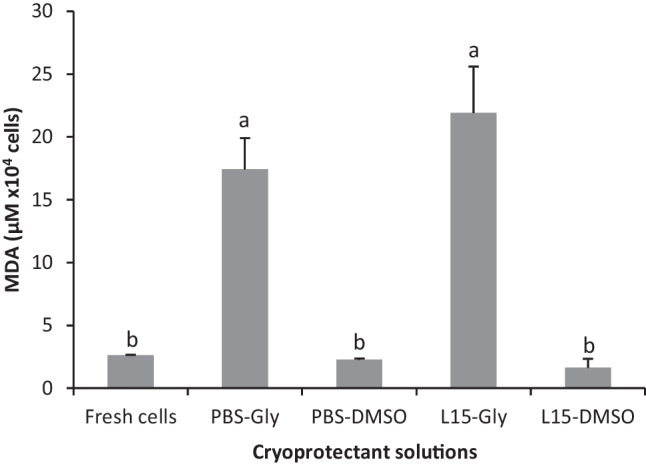
Malondialdehyde (MDA) levels determined in testes fragments cryopreserved in PBS or L-15 media incorporating DMSO or glycerol. Significant differences between treatments are signed by different letters (mean values ± SD, *n*=10, *p* ≤ 0.05)

The analysis of lipid peroxidation indicated no differences between fresh cells and those frozen with DMSO in either PBS or L-15. However, an important and significant increase in oxidation was detected in samples containing glycerol as a cryoprotectant (Fig. 5).

**Fig. 6:**
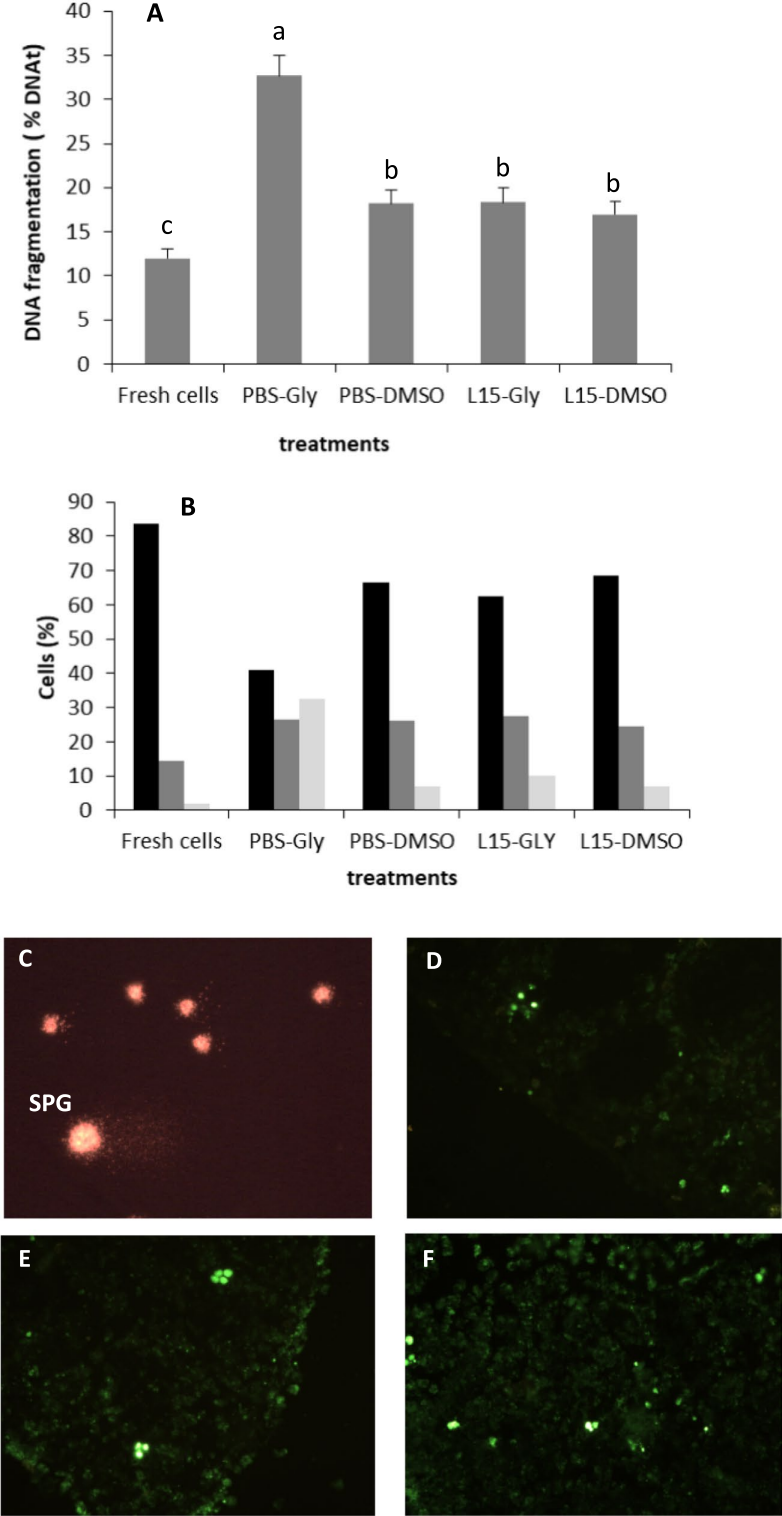
DNA fragmentation in fresh and cryopreserved spermatogonia (**A**) determined using the comet assay method as shown by the micrograph (**C**). Percentage of spermatogonia with different degrees of DNA damage: low 0–20%, medium 20–40%, and high >40% (**B**). A total of 800–1000 cells were analyzed per treatment. Significant differences between treatments are signed by different letters (mean values ± SD, *n*=10, *p* ≤ 0.05). Micrographs of TUNEL assay (apoptosis) performed in frozen/thawed testes fragments cryopreserved with PBS-glycerol (**D**) and L15-DMSO (**E**). The right micrograph represents fresh testes processed for the same technique (**F**)

The analysis of DNA fragmentation in thawed germ cells revealed an increase in the percentage of DNA_t_ in all thawed cells in comparison with those obtained from fresh testes. Mean DNA_t_ increased from 11.95 ± 1.08% in fresh samples to values higher than 16% in all treatments. Samples frozen in PBS + glycerol reached mean values of 32.69 ± 2.27%, significantly higher than the other treatments (Fig. 6A–B). This treatment also provided the highest percentage of germ cells with more than 40% of their DNA fragmented, which is considered to be a high level of damage, and non-compatible with long-term cell functionality (Fig. 6C). In fresh samples, only 2.1% of spermatogonia presented high levels of DNA damage, followed by samples cryopreserved with L15 + DMSO, PBS + DMSO, and L15 + glycerol (6.9%, 7.2%, 10.0%, respectively) (Fig. 6C).

A small number of cells were identified as apoptotic cells using the TUNNEL assay, without any differences between treatments and fresh testes fragments. Apoptotic cells were located in small groups within the same testicular cyst (Fig. 6D–F).

**Fig 7:**
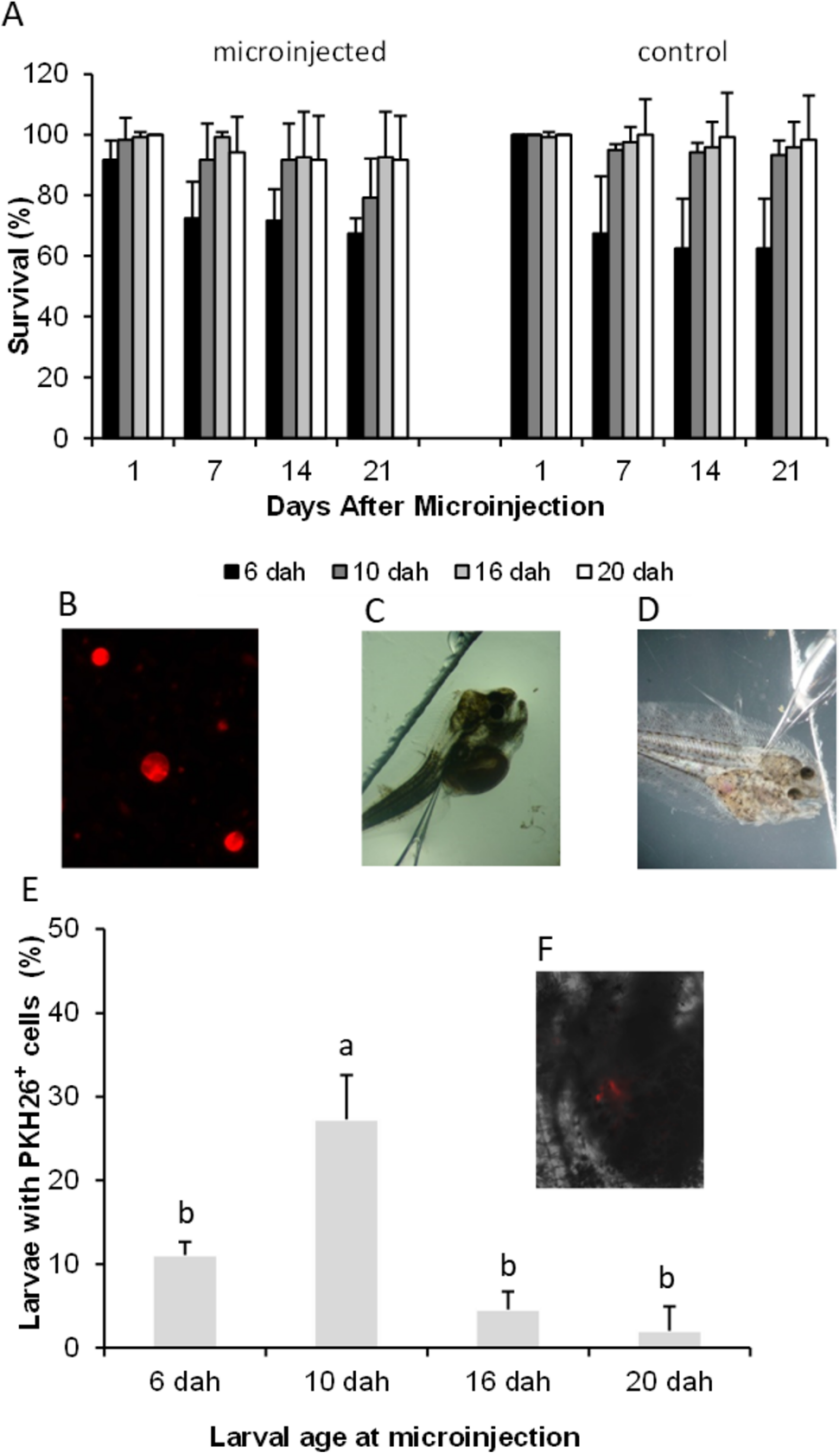
Survival rate of control and microinjected larvae with (13.4 nL) (**A**). Micrographs of PKH26 marked germ cells (**B**) and two larval stages used for microinjection: the beginning of larval metamorphosis at 10 dah (**C**) and the end of metamorphosis at 16 dah (**D**). Percentage of larvae incorporating spermatogonia-marked cells after three weeks of microinjection (**E**). Micrograph of microinjected larvae incorporating PKH26 marked germ cells. The illustration shows a fluorescence image overlapping with brightfield image (**F**). Significant differences between treatments are signed by different letters (mean values ± SD, *n*=120, *p* ≤ 0.05)

The survival rate in *Solea senegalensis* larvae decreased by approximately 20% in 6 dah larvae from day 1 to day 7 after microinjection, affecting both microinjected and control larvae (Fig. 7A). No decrease in survival rates was noticed in larvae microinjected at later stages, being similar to those in the control batches. Regarding the use of cell transplantation procedures as a functional test, we have seen that Senegalese sole germ cells were positively labeled with PKH26 as shown in Fig. 7B and microinjection was successfully performed in the intraperitoneal cavity of larvae at all the tested stages (Fig. 7C–D). Three weeks after cell transplantation, the presence of transplanted cells was observed in the abdominal region of the recipient larvae (Fig. 7F), but not in the controls, showing that larvae microinjected at the beginning of metamorphosis (10 dah) had a significantly higher capacity to incorporate post-thaw germ cells (27.30 ± 5.27%) (Fig. 7E) than other stages (lower than 11%).

## Discussion

The present study has demonstrated for the first time that fish testes fragments can be successfully cryopreserved in the flatfish Senegalese sole, an important aquaculture species, maintaining germ cell viability, functionality, and the capacity for incorporation into recipient larvae.

Cryopreservation is a very useful technique for xeno and allo-transplantation studies in fish and has previously been applied in rainbow trout primordial germ cells (PGCs) and spermatogonia suspensions, in Nile tilapia germ cell suspensions (Lacerda et al. 2010; Kobayashi et al. 2007; Yoshizaki et al. 2011), in starry goby, *Asterropteryx semipunctata* germ cells (Hagedorn et al. 2018), within others. Our results showed that the efficiency of cryopreserving isolated cells in suspension was lower in terms of cell integrity than cryopreserving testes fragments, regardless of the protocol used. This fact could be associated with cell manipulation procedures involved in cell dissociation and isolation (e.g., trypsinization), which render the cells more sensitive to cryoinjuries during freezing. Similar results were obtained by Hagedorn et al. (2018) when freezing single-cell suspensions against gonadal tissue from the Indo-Pacific marine starry goby. Freezing testes fragments is simpler than dissociating and freezing cell suspensions, facilitating the storage of germ cells under different conditions of work, such as offshore in the sea or in fish farms, as was our case. Riesco et al. (2012) working with zebrafish genital ridges containing PGCs also demonstrated better results in terms of cell viability and DNA integrity in fragments frozen in cryovials or straws than in PGC suspensions encapsulated in microspheres. Therefore, the cryopreservation of testes fragments containing undifferentiated germ cells seems to be a better option for further cell transplantation. This technique has been attempted in some mammalian species (Izadyar et al. 2002; Keros et al. 2007; Redden et al. 2009; Whelan et al. 2022), transplanting the fragments directly into the host after thawing. However, in fish, transplantation is performed with isolated cells, making it necessary to dissociate the tissue after thawing. We reported high numbers of recovered spermatogonia after freezing/thawing and dissociation. DMSO protected more specifically the testicular cells since a significantly higher number of spermatogonia were recovered with treatments incorporating this cryoprotectant, especially combined with L-15 media.

It is well known that cryopreservation can produce different types of cell damage, affecting cell integrity and DNA stability due, at least partially, to oxidative stress, as reviewed by Aitken and Drevet (2020). This seems to be extremely important in germ cells since after thawing they need to maintain their capacity to differentiate into other germ cells and proliferate to maintain male fertility throughout their lifetime (Ehmcke et al. 2006).

Cells obtained by dissociation of thawed testes fragments showed no significant differences between treatments, regarding plasma membrane integrity; however, a slight reduction in esterase activity was detected by calcein incubation of germ cells cryopreserved with L-15 media, regardless of the used cryoprotectant. It has been largely reported that chilling injury can modify the structure and integrity of membranes, mainly composed of phospholipids and cholesterol, because during cryopreservation the cooling process may cause phase transition of membrane lipids, impair membrane protein function, and produce loss of these compounds (Beirão et al. 2012; Di Santo et al. 2012). Our protocols promote a reduction in membrane integrity but seemed to provide enough cells with the required protection to be used for further transplantation since 57% of viable cells were recovered from PBS + 1.5 M DMSO treatment. Our cell viability data are encouraging since other authors working with meagre (*Argyrosomus regius*) spermatogonia obtained 29% of cell survival using 1.4 M DMSO (Zupa et al. 2020) or more variable results in zebrafish spermatogonia depending on the used DMSO concentration (15%, 20%, and 60% for 1.6 M, 1 M and 1.3 M, respectively) (Marinović et al. 2019). In the present study, we did not test different concentrations of DMSO, but according to previous data by Marinović et al. (2019), this should be considered in future studies.

Cryopreservation has also been shown to diminish the antioxidant activity of cells, increasing the risk of oxidative stress. In addition, a modification of mitochondrial membrane fluidity may also lead to an alteration of its potential and release of reactive oxygen species (ROS), responsible for oxidative damage (Aitken and Drevet 2020; Said et al. 2010). In our study, the analysis of lipid peroxidation indicated no differences between fresh cells and those frozen with DMSO in either PBS or L-15. However, an important and significant increase in oxidation was detected in samples containing glycerol as a cryoprotectant. It seems that, although it has been proposed as a good cryoprotectant for certain mammalian cell types, glycerol produces oxidative stress in these germ cells which could affect DNA stability and impair other cell functions (Aitken and Drevet 2020). Spermatogonia are more resistant to ROS damage than other germ cells (e.g., spermatozoa) due to their better protective antioxidant system (Celino et al. 2011; Paul et al. 2009; Zhu et al. 2022), but this mechanism may be affected during cryopreservation, making cells more susceptible to ROS damage. In fact, we investigated this hypothesis in the present study and observed an increase in DNA fragmentation in germ cells from thawed testes fragments compared with those from fresh samples (16.95–32.69% DNA_t_ *vs* 11.95% DNA_t_). The treatment with PBS+glycerol gave a significantly higher number of cells with fragmented DNA and also a higher degree of fragmentation of those cells, confirming the results obtained by lipid peroxidation. DNA damage could be associated with other causes rather than ROS, since as we previously pointed out, spermatogonia are less sensitive to oxidative damage than other germ cells, but they are more prone to external agents inducing DNA damage. This is in part due to the lower level of chromatin condensation and to their location on the unprotected side of the testis barrier, being more exposed to circulating molecular damaging factors than cells on the protected side. This may be especially important during cryopreservation when high concentrations of cryoprotectants are used to allow correct preservation of all cells inside the testes fragment, spermatogonia being exposed to a more toxic solution for longer periods. The consequences of DNA damage in spermatogonia could have severe implications. In teleost, differentiated spermatogonia are reported to undergo mitosis 6 to 16 times (Nóbrega et al. 2009; Shulz et al. 2010), followed by two consecutive cycles of meiosis, and therefore, any damage that could not be repaired by the DNA repair mechanisms may be transported to the following generations in the germ line, producing aberrant spermatozoa, and thus impairing fertilization ability. Therefore, DNA integrity is critical for these stem cells, and a correct cryopreservation protocol needs to ensure the integrity of SPG genes required for gamete development and fertilization. The apoptotic process did not seem to be significant in the testes, and the distribution of apoptotic cells in particular cysts seemed to be more related to a physiological process than to secondary effects produced by freezing injury since they were also present in fresh samples.

Our cryopreservation protocol incorporating L-15 + DMSO produced the best results in terms of spermatogonia recovery and quality. Although there was a slight increase in DNA fragmentation compared to fresh samples, especially in higher levels of DNA damage, the results obtained using this protocol showed that more than half of the spermatogonia had less than 20% fragmentation, providing a good supply of undamaged cells to be used in further transplantation.

Our experiments on germ cell transplantation into Senegalese sole larvae confirmed the findings regarding the quality of L-15 + DMSO cryopreserved cells, since transplantation was successfully achieved, and 3 weeks after treatment, larvae showed incorporation of labeled spermatogonia. In a previous study, we demonstrated that important changes in the expression pattern of different *vasa* gene transcripts take place in this species from hatching to post-metamorphic life (after 24 dah), indicating changes in germ cell activity during the metamorphic process. In post-metamorphic larvae, the gonad *primordium* containing undifferentiated germ cells initiates a considerable proliferation process, corresponding probably to the high levels of *vasa3-4* expression from 24 dah onwards (Pacchiarini et al. 2013). Consequently, the analyzed period (3 weeks after microinjection) was considered the most appropriate to detect whether incorporated germ cells would be present or not. The percentage of larvae incorporating transplanted germ cells (PKH26 positive cells) was significantly higher when 10 dah microinjected larvae were used as recipients than at other ages (6, 16, and 20 dah). As demonstrated by Takeuchi et al. (2009) in the nibe croaker, there is a labile period for the incorporation of undifferentiated germ cells into the gonad *primordium*, which varies between species due to their different development characteristics. This period corresponds to the moment when these microinjected cells receive external stimuli and migrate into the surrounding area, adhering to the peritoneal wall or to the dorsal mesentery of the recipient organism (Takeuchi et al. 2009).

Senegalese sole larvae spend much energy on important transformations during the metamorphosis process related to the final stage of organogenesis, where organs migrate inside the body cavity, the intestine elongates to adapt to the new body structure, and the right eye migrates to the upper part of the body, going from a pelagic life to a benthonic stage (Sarasquete et al. 2019). Our data demonstrate that it is also during this period of life that Senegalese sole has the capacity to receive allogenic cells. In our study, larvae larger than 5.2 mm (10 dah) showed a significant decrease in their ability to incorporate germ cells. Although endogenous germ cells would be at their stage of proliferation when the larvae were analyzed (3 weeks after microinjection), the optimum threshold for exogenous cell transplantation and incorporation was overpassed at the moment of microinjection. Therefore, microinjection of larvae at 20 dah was only succeeded by 3%. Moreover, at approximately 9 to 11 dah, Senegalese sole starts to develop the immune system, firstly by the appearance of thymus and some undifferentiated cells (Sarasquete et al. 2019), possibly eliciting some immune reaction contributing to the worse results observed at later ages. Similar findings were reported by Takeuchi et al. (2009) in nibe croaker, where 4-mm recipients (6 dah) were more efficient receptors than larvae of other sizes or ages. In the present study, Senegalese sole proved to be a good model for flatfish transplantation studies since unlike other marine reported species (yellowtail 10% survival rate, nibe croaker 20.6% survival rate after 3 weeks), the survival rates at the optimal period for transplantation were high (10 dah larvae 98.3% and 79.16%, 1 and 21 days after transplantation, respectively) and did not significantly differ from the controls. Therefore, although our incorporation rates were lower (28%), probably because of the use of cryopreserved cells than the values recorded by Morita et al. (2012) for yellowtail (100%), the survival rates compensate for this result, giving higher efficiency rates (incorporation rate × survival rate) at the end of the experiment than those reported for other marine species (10% in yellowtail and 7.5% in nibe croaker) (Morita et al. 2012; Takeuchi et al. 2009).

Our results are very promising and open new perspectives in the reproduction of Senegalese sole including surrogate production with the use of cryopreserved germ cells transplanted into an appropriate host species.

## Supplementary information

ESM 1 (DOCX 514 kb)

## Data Availability

All data generated or analyzed during this study are included in this published article.

## References

[CR1] Aitken RJ, Drevet JR (2020) The importance of oxidative stress in determining the functionality of mammalian spermatozoa: a two-edged sword. Antioxidants 9(2): 111; 10.3390/antiox9020111 10.3390/antiox9020111 PMC7070991 32012712

[CR2] Beirão J, Zilli L, Vilella S, Cabrita E, Schiavone R, Herráez MP (2012) Improving sperm cryopreservation with antifreeze proteins: effect on gilthead seabream (*Sparus aurata*) plasma membrane lipids. Biol Reprod 86(59):1–9. 10.1095/biolreprod.111.093401 10.1095/biolreprod.111.093401 22088915

[CR3] Cabrita E, Pacchiarini T, Sarasquete C, Herráez MP (2010) Development of a cryopreservation protocol for Senegalese sole testicular germ cells. Cryobiology 61(3):405. 10.1016/j.cryobiol.2010.10.145

[CR4] Cabrita E, Robles V, Rebordinos L, Sarasquete C, Herráez MP (2005) Evaluation of DNA damage in rainbow trout (*Oncorhynchus mykiss*) and gilthead sea bream (S*parus aurata*) cryopreserved sperm. Cryobiology 50:144–153. 10.1016/j.cryobiol.2004.12.003 15843004 10.1016/j.cryobiol.2004.12.003

[CR5] Celino FT, Yamaguchi S, Miura C, Ohta T, Tozawa Y, Iwai T, Miura T (2011) Tolerance of spermatogonia to oxidative stress is due to high levels of Zn and Cu/Zn superoxide dismutase. PLoS One 6:e16938. 10.1371/journal.pone.0016938 21364994 10.1371/journal.pone.0016938 PMC3041797

[CR6] de Siqueira-Silva DH, dos Santos Silva AP, da Silva CR, Senhorini A, Ninhaus-Silveira A, Veríssimo-Silveira R (2021) Preliminary study on testicular germ cell isolation and transplantation in an endangered endemic species *Brycon orbignyanus* (Characiformes: Characidae). Fish Physiol Biochem 47:767–776. 10.1007/s10695-019-00631-8 30937624 10.1007/s10695-019-00631-8

[CR7] Di Santo M, Tarozzi N, Nadalini M, Borini A (2012) Human sperm cryopreservation: update on techniques, effect on DNA integrity, and implications for ART. Adv Urol 854837. 10.1155/2012/854837 10.1155/2012/854837 PMC3238352 22194740

[CR8] Ehmcke J, Wistuba J, Schlatt S (2006) Spermatogonial stem cells: questions, models and perspectives. Hum Reprod Update 12:275–282. 10.1093/humupd/dmk001 16446319 10.1093/humupd/dmk001

[CR9] Fernández-Díez C, Pérez-Sanchiz R, Sarasquete C, Cabrita E, Herráez MP (2012) New tools for genome preservation: grafting spermatogonia in brown trout (*Salmo trutta*). J Appl Ichthyol 28(6):916–918. 10.1111/jai.12077

[CR10] Franek R, Cheng Y, Fuckova M, Kaspar V, Xie X, Shah MA, Linhart O, Sauman I, Psenicka M (2022) Who is the best surrogate for germ stem cell transplantation in fish? Aquaculture 549:737759

[CR11] Franek R, Marinović Z, Lujić J, Urbanyi B, Fuckova M, Kaspar V, Psenicka M, Horváth A (2019) Cryopreservation and transplantation of common carp spermatogonia. PLoS One 14(4):e0205481. 10.1371/journal.pone.0205481 30998742 10.1371/journal.pone.0205481 PMC6472724

[CR12] Hagedorn MM, Daly JP, Carter VL, Cole KS, Jaafar Z, Lager CVA, Parenti LR (2018) Cryopreservation of fish spermatogonial cells: the future of natural history collections. Sci Rep 8:6149. 10.1038/s41598-018-24269-3 29670253 10.1038/s41598-018-24269-3 PMC5906666

[CR13] Izadyar I, Matthijs-Rijsenbilt JJ, den Ouden K, Creemers LB, Woelders H, de Rooij DJ (2002) Development of a cryopreservation protocol for type A spermatogonia. J Androl 23:537–545. 10.1002/j.1939-4640.2002.tb02276.x 12065461

[CR14] Kaya T, Torisawa Y-S, Oyamatsu D, Nishizawa M, Matsue T (2003) Monitoring the cellular activity of a cultured single cell by scanning electrochemical microscopy (SECM). A comparison with fluorescence viability monitoring. Biosens Bioelectron 18:1379–1383. 10.1016/S0956-5663(03)00083-6 12896839 10.1016/s0956-5663(03)00083-6

[CR15] Keros V, Hultenby K, Borgstrom B (2007) Methods of cryopreservation of testicular tissue with viable spermatogonia in pre-pubertal boys undergoing gonadotoxic cancer treatment. Hum Reprod 22:1384–1392. 10.1093/humrep/del508 17259225 10.1093/humrep/del508

[CR16] Kobayashi T, Takeuchi Y, Takeuchi T, Yoshizaki G (2007) Generation of viable fish from cryopreserved primordial germ cells. Mol Reprod Develop 74:207–213. 10.1002/mrd.20577 10.1002/mrd.20577 16998845

[CR17] Lacerda S, Costa J, Campos-Junior P, Segatelli T, Takeuchi Y, Yazawa R, Morita T, Yoshizaki G, França L (2013) Germ cell transplantation as a potential biotechnological approach to fish reproduction. Fish Physiol Biochem 39(1):3–11. 10.1007/s10695-012-9606-4 22290474 10.1007/s10695-012-9606-4

[CR18] Lacerda SMS, Batlouni SR, Costa GMJ, Segatelli TM, Quirino BR, Queiroz BM, Kalapothakis E, França LR (2010) A new and fast technique to generate offspring after germ cells transplantation in adult fish: the Nile tilapia (*Oreochromis niloticus*) model. PLoS One 5:e10740. 10.1371/journal.pone.0010740 20505774 10.1371/journal.pone.0010740 PMC2873995

[CR19] Marinović Z, Li Q, Lujic J, Iwasaki Y, Csenki Z, Urbányi B, Yoshizaki G, Horváth Á (2019) Preservation of zebrafish genetic resources through testes cryopreservation and spermatogonia transplantation. Sci Rep 9:13861. 10.1038/s41598-019-50169-1 31554831 10.1038/s41598-019-50169-1 PMC6761286

[CR20] Martínez-Páramo S, Diogo P, Dinis MT, Herráez MP, Sarasquete C, Cabrita E (2012) Incorporation of ascorbic acid and α-tocopherol to the extender media to enhance antioxidant system of cryopreserved seabass sperm. Theriogenology 77:1129–1136. 10.1016/j.theriogenology.2011.10.017 22153272 10.1016/j.theriogenology.2011.10.017

[CR21] Morais S, Aragão C, Cabrita E, Estevez A, Yúfera M, Valente LMP, Conceição LEC, Dias J, Engrola S, Gisbert E, Costas B, Constenla M, Mañanós E, Duncan N, Dinis MT (2016) New developments and biological insights into the farming of *Solea senegalensis* reinforcing its aquaculture potential. Rev Aquac 6:1–37. 10.1111/raq.12091

[CR22] Morita T, Kumakura N, Morishima K, Mitsuboshi T, Ishida M, Hara T, Kudo S, Miwa M, Ihara S, Higuchi K, Takeuchi Y, Yoshizaki G (2012) Production of donor-derived offspring by allogeneic transplantation of spermatogonia in the yellowtail (*Seriola quinqueradiata*). Biol Reprod 86(6):176, 1–11. 10.1095/biolreprod.111.097873 10.1095/biolreprod.111.097873 22460666

[CR23] Nóbrega RH, Batlouni SR, França LR (2009) An overview of functional and stereological evaluation of spermatogenesis and germ cell transplantation in fish. Fish Physiol Biochem 35:197–206. 10.1007/s10695-008-9252-z 18716890 10.1007/s10695-008-9252-z

[CR24] Nóbrega RH, Greebe CD, van de Kant H, Bogerd J, França LR, Schulz RW (2010) Spermatogonial stem cell niche and spermatogonial stem cell transplantation in zebrafish. PLoS One 5(9):e12808. 10.1371/journal.pone.0012808 20862221 10.1371/journal.pone.0012808 PMC2942835

[CR25] Pacchiarini T, Cross I, Leite R, Gavaia P, Ortiz-Delgado JB, Pousão-Ferreira P, Rebordinos L, Sarasquete C, Cabrita E (2013) The *Solea senegalensis vasa* transcripts: molecular characterization, tissue distribution and developmental expression profiles. Reprod Fert Develop 25:646–660. 10.1071/RD11240 10.1071/RD11240 22954189

[CR26] Pacchiarini T, Sarasquete C, Cabrita E (2014) Development of interspecies testicular germ cells transplantation in flatfish. Reprod Fertil Dev 26:690–702. 10.1071/RD13103 23735683 10.1071/RD13103

[CR27] Paul C, Teng S, Saunders PTK (2009) A single, mild, transient scrotal heat stress causes hypoxia and oxidative stress in mouse testes, which induces germ cell death. Biol Reprod 80:913–919. 10.1095/biolreprod.108.071779 19144962 10.1095/biolreprod.108.071779 PMC2709966

[CR28] Psenicka M, Saito T, Linhartová Z, Gazo I (2015) Isolation and transplantation of sturgeon early-stage germ cells. Theriogenology 83(6):1085–1092. 10.1016/j.theriogenology.2014.12.010 25559841 10.1016/j.theriogenology.2014.12.010

[CR29] Redden E, Davey R, Borjigin U, Hutton K, Hinch G, Hope S, Hill J, Herrid M (2009) Large quantity cryopreservation of bovine testicular cells and its effect on enrichment of type A spermatogonia. Cryobiology 58:190–195. 10.1016/j.cryobiol.2008.12.005 19138683 10.1016/j.cryobiol.2008.12.005

[CR30] Riesco MF, Martinez-Pastor F, Chereguini O, Robles V (2012) Evaluation of zebrafish (*Danio rerio*) PGCs viability and DNA damage suing different cryopreservation protocols. Theriogenology 77:122–130. 10.1016/j.theriogenology.2011.07.024 21872308 10.1016/j.theriogenology.2011.07.024

[CR31] Rivers N, Daly J, Jones R, Temple-Smith P (2020a) Cryopreservation of testicular tissue from Murray River Rainbowfish, Melanotaenia fluviatilis. Sci Rep. 10:19355. 10.1038/s41598-020-76378-7 33168894 10.1038/s41598-020-76378-7 PMC7653925

[CR32] Rivers N, Daly J, Jones R, Temple-Smith P (2020b) New directions in assisted breeding techniques for fish conservation. Reprod Fertil Dev 32:807–821. 10.1071/RD19457 32527372 10.1071/RD19457

[CR33] Said TM, Gaglani A, Agarwal A (2010) Implication of apoptosis in sperm cryoinjury. Reprod BioMed Online 21(4):456–462. 10.1016/j.rbmo.2010.05.011 20800544 10.1016/j.rbmo.2010.05.011

[CR34] Sarasquete C, Gisbert E, Ortiz-Delgado JB (2019) Embryonic and larval ontogeny of the Senegalese sole, *Solea senegalensis* in: Muñoz-Cueto JA, Mañanós EL, Sanchez-Vasquez FJ (Eds). The biology of sole, CRC press, Taylor and Francis, Boca Raton, USA, 216-262.

[CR35] Schlatt S (2002) Spermatogonial stem cell preservation and transplantation. Mol Cell Endocrinol 187:107–111 11988317 10.1016/s0303-7207(01)00706-7

[CR36] Shulz RW, de França LR, Lareyre JJ, LeGac F, Chiarini-Garcia H, Nóbrega RH, Miura T (2010) Spermatogenesis in fish. Gen Comp Endocrinol 165:390–411. 10.1016/j.ygcen.2009.02.013 19348807 10.1016/j.ygcen.2009.02.013

[CR37] Takeuchi Y, Higuchi K, Yatabe T, Miwa M, Yoshizaki G (2009) Development of spermatogonial cell transplantation in Nibe croaker, *Nibea mitsukurii* (Perciformes, Sciaenidae). Biol Reprod 81:1055–1063. 10.1016/j.ygcen.2009.02.013 19605788 10.1095/biolreprod.109.077701

[CR38] Takeuchi Y, Yoshizaki G, Takeuchi T (2004) Surrogate broodstock produces salmonids. Nature 430(7000):629–630. 10.1038/430629a 15295587 10.1038/430629a

[CR39] Whelan EC, Yang F, Avarbock MR, Sullivan MC, Beiting DP, Brinster RL (2022) Reestablishment of spermatogenesis after more than 20 years of cryopreservation of rat spermatogonial stem cells reveals an important impact in differentiation capacity. PLoS Biol 20(5):e3001618. 10.1371/journal.pbio.3001618 35536782 10.1371/journal.pbio.3001618 PMC9089916

[CR40] Xie X, Nóbrega R, Pšenička M (2020) Spermatogonial stem cells in fish: characterization, isolation, enrichment, and recent advances of *in vitro* culture systems. Biomol 10:644. 10.3390/biom10040644 10.3390/biom10040644 PMC7226347 32331205

[CR41] Yazawa R, Kubokawa T, Ichida K, Kawamura W, Tani R, Kamio S, Morita T, Yoshizaki G (2021) Establishment of a tracing technique for transplanted bluefin tuna germ cells in recipient’s gonads using monoclonal antibodies specifically recognizing bluefin tuna spermatogenic cells. Fish Sci 87:105–112. 10.1007/s12562-020-01486-2

[CR42] Ye H, Li C, Yue H-M, Du H, Yang X-G, Yoshino T, Hayashida T, Takeuchi Y, Wei Q-W (2017) Establishment of intraperitoneal germ cell transplantation for critically endangered Chinese sturgeon *Acipenser sinensis*. Theriogenology 94:37–47. 10.1016/j.theriogenology.2017.02.009 28407859 10.1016/j.theriogenology.2017.02.009

[CR43] Yoshizaki G, Fujinuma K, Iwasaki Y, Okutsu T, Shikina S, Yazawa R, Takeuchi Y (2011) Spermatogonial transplantation in fish: a novel method for the preservation of genetic resources. Comp Biochem Physiol D 6:55–61. 10.1016/j.cbd.2010.05.003 10.1016/j.cbd.2010.05.003 20541987

[CR44] Zhou L, Wang X, Liu Q, Yang J, Xu S, Wu Z, Wang Y, You F, Song Z, Li J (2021) Successful spermatogonial stem cells transplantation within pleuronectiformes: first breakthrough at inter-family level in marine fish. Int J Biol Sci 17(15):4426–4441. 10.7150/ijbs.63266 34803508 10.7150/ijbs.63266 PMC8579436

[CR45] Zhu X, Yu C, Wu W, Shi L, Jiang C, Wang L, Ding Z, Liu Y (2022) Zinc transporter ZIP12 maintains zinc homeostasis and protects spermatogonia from oxidative stress during spermatogenesis. Reprod Biol Endocrinol 20:17. 10.1186/s12958-022-00893-7 35065654 10.1186/s12958-022-00893-7 PMC8783530

[CR46] Zupa R, Martino NA, Marzano G, Dell’Aquila ME, Corriero A (2020) Meagre *Argyrosomus regius* (Asso, 1801) Stem spermatogonia: histological characterization, immunostaining, *In Vitro* Proliferation, and Cryopreservation. Animals 10:851. 10.3390/ani10050851 32423131 10.3390/ani10050851 PMC7278407

