# Supplementary figures and images for: Post-thaw quality assessment of testicular fragments as a source of spermatogonial cells for surrogate production in the flatfish Solea senegalensis
Source: Fish Physiol Biochem. 2023 Aug 29;50(5):1971–85. doi: 10.1007/s10695-023-01232-2 (PMC11576765; doi:10.1007/s10695-023-01232-2)

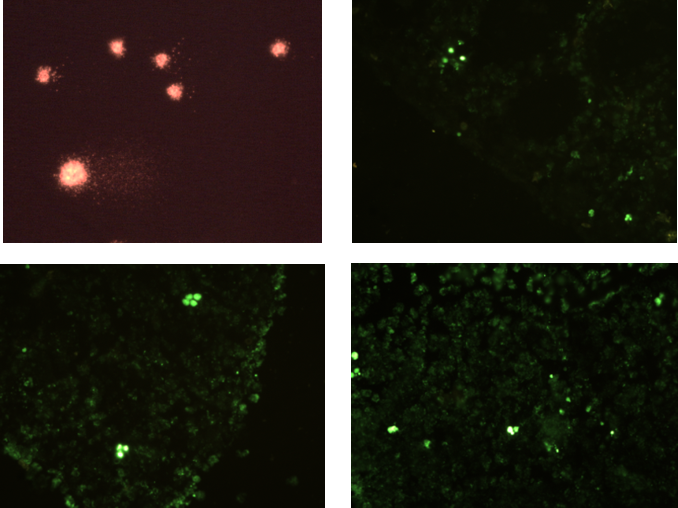

Supplement: Supplementary file 1 — (DOCX 514 kb) [file 10695_2023_1232_MOESM1_ESM.docx]
